# The Urinary Bladder is Rich in Glycosphingolipids Composed of Phytoceramides

**DOI:** 10.1016/j.jlr.2022.100303

**Published:** 2022-10-27

**Authors:** Takashi Watanabe, Akemi Suzuki, Shin Ohira, Shinji Go, Yuta Ishizuka, Takuya Moriya, Yoshiyuki Miyaji, Tota Nakatsuka, Keita Hirata, Atsushi Nagai, Junko Matsuda

**Affiliations:** 1 Department of Pathophysiology and Metabolism, Kawasaki Medical School, Kurashiki, Okayama, Japan; 2 Institute of Molecular Biomembrane and Glycobiology, Tohoku Medical and Pharmaceutical University, Sendai, Miyagi, Japan; 3 Department of Urology, Kawasaki Medical School, Kurashiki, Okayama, Japan; 4 Department of Pathology, Kawasaki Medical School, Kurashiki, Okayama, Japan

**Keywords:** sphingolipids, ceramides, 20-carbon long-chain base, 2-hydroxy fatty acid, DES2, FA2H, SPTLC3, SPTSSB, epithelial cell, urothelial cell, BCG, Bacille Calmette-Guerin, cDNA, complementary DNA, CERS, ceramide synthase, DES1, dihydroceramide:sphinganine Δ4-desaturase, DES2, dihydroceramide:sphinganine C4-hydroxylase, Epi, epithelial layer, FA2H, fatty acid 2-hydroxylase, GalCer, galactosylceramide, Gb3Cer, globotriaosylceramide, Gb4Cer, globotetraosylceramide, GD3, Neu5Ac-Neu5Ac-Gal-Glc-Ceramide, GlcCer, glucosylceramide, GM1, Gal-GalNAc-(Neu5Ac-)Gal-Glc-Ceramide, GM3, Neu5Ac-Gal-Glc-Ceramide, GSL, glycosphingolipid, HexCer, hexosylceramide, HPTLC, high-performance TLC, LacCer, lactosylceramide, LCB, long-chain base, MRM, multiple reaction monitoring, qRT-PCR, quantitative RT-PCR, SPT, serine palmitoyltransferase, *SPTLC1*, serine palmitoyltransferase LCB subunit 1, *SPTLC2*, serine palmitoyltransferase LCB subunit 2, *SPTLC3*, serine palmitoyltransferase LCB subunit 3, *SPTSSA*, serine palmitoyltransferase small subunit A, *SPTSSB*, serine palmitoyltransferase small subunit B, SubEpi, subepithelial layer

## Abstract

Glycosphingolipids (GSLs) are composed of a polar glycan chain and a hydrophobic tail known as ceramide. Together with variation in the glycan chain, ceramides exhibit tissue-specific structural variation in the long-chain base (LCB) and *N*-acyl chain moieties in terms of carbon chain length, degree of desaturation, and hydroxylation. Here, we report the structural variation in GSLs in the urinary bladders of mice and humans. Using TLC, we showed that the major GSLs are hexosylceramide, lactosylceramide, globotriaosylceramide, globotetraosylceramide, Neu5Ac-Gal-Glc-Ceramide, and Neu5Ac-Neu5Ac-Gal-Glc-Ceramide. Our LC-MS analysis indicated that phytoceramide structures with a 20-carbon LCB (4-hydroxyeicosasphinganine) and 2-hydroxy fatty acids are abundant in hexosylceramide and Neu5Ac-Gal-Glc-Ceramide in mice and humans. In addition, quantitative PCR demonstrated that *DES2* and *FA2H*, which are responsible for the generation of 4-hydroxysphinganine and 2-hydroxy fatty acid, respectively, and *SPTLC3* and *SPTSSB*, which are responsible for the generation of 20-carbon LCBs, showed significant expressions in the epithelial layer than in the subepithelial layer. Immunohistochemically, dihydroceramide:sphinganine C4-hydroxylase (DES2) was expressed exclusively in urothelial cells of the urinary bladder. Our findings suggest that these ceramide structures have an impact on membrane properties of the stretching and shrinking in transitional urothelial cells.

Glycosphingolipids (GSLs) are one of the membrane lipids in mammalian cells. They are involved in a wide spectrum of cellular functions, such as growth, adhesion, migration, and death (1). Defects in GSL metabolism are associated with, for example, lysosomal sphingolipid storage diseases, neurodegenerative diseases such as Alzheimer’s and Parkinson’s diseases, and cancers (2). GSLs are composed of a polar head glycan chain and a hydrophobic tail known as ceramide. The glycan chains and ceramides show highly diverse and strictly regulated structural variations among tissues (3, 4, 5). The variations of ceramides reside in carbon chain length, degree of desaturation, and hydroxylation of the *N*-acyl chain and long-chain base (LCB). Six distinct ceramide synthases (CERs) encoded by *CERS1* to *CERS6* produce variation in *N*-acyl carbon chain length (6, 7). Fatty acid 2-hydroyxlase (encoded by *FA2H*) catalyzes the production of 2-hydroxy fatty acids of sphingolipids in myelin (8, 9). Serine palmitoyltransferases (SPTs) are heterotrimeric proteins consisting of a large subunit dimer and one of two small subunits and responsible for the variation in carbon-chain length in LCBs (10). Dihydroceramide:sphinganine Δ4-desaturase (DES1) produces ceramides with sphingenine, which are the most abundant LCBs in mammalian cells (11). Dihydroceramide:sphinganine C4-hydroxylase (DES2) produces phytoceramides with an additional hydroxy group at sphinganine C-4, which are abundant LCBs in the small intestine and kidney (12). However, the biological roles of the above species are unclear.

The GSL composition in the small intestines of mice is developmentally regulated (5). The ceramide moieties of GSLs are mainly composed of phytosphingosine and 2-hydroxy fatty acid from the neonatal period to adulthood, and the polar glycan head group of GSLs is converted at around 2 weeks of age from glucosylceramide (GlcCer), Neu5Ac-Gal-Glc-Ceramide (GM3), Gal-GalNAc-(Neu5Ac-)Gal-Glc-Ceramide, and Neu5Ac-Gal-GalNAc-(Neu5Ac-)Gal-Glc-Ceramide into GlcCer and Gal-GalNAc-Gal-Glc-Ceramide (asialo GM1), which is synchronized with the expression of nutrient transporters. These results suggest the importance of GSL structures for modulating the membrane properties of intestinal epithelial cells.

Urothelial cells line the urinary tract, including the renal pelvis, ureters, and urinary bladder, and are known as a transitional epithelium because they are composed of superficial, intermediate, and basal layers (13, 14). These unique epithelial structures enable changes in urinary bladder volume depending on the accumulation and release of urine. Characteristically, terminally differentiated superficial cells called umbrella cells are large dome-shaped polyhedral cells that change their morphology according to urine volume from cuboidal to highly stretched. To enable these changes, it is speculated that the apical membrane of urothelial cells has unique protein and lipid compositions. Indeed, the apical membrane of umbrella cells is covered with highly specialized two-hexagonally packed 16 nm protein plaques composed of a transmembrane complex of uroplakins (15, 16). No study has focused on the membrane lipids of urinary bladder tissues.

In this study, we hypothesized that urothelial cells have unique GSL compositions to enable their stretching-and-shrinking membrane properties and investigated the structural diversity of GSLs in urinary bladder tissues of mice and humans.

## Materials and Methods

### Urinary bladder tissues of mice and humans

Whole urinary bladder tissues were collected from male and female wild-type mice of the C57BL/6J background at the age of 120 days. The tissues were immediately frozen in liquid nitrogen and stored at −80°C until use for glycolipid or RNA extraction. For histopathological analysis, the tissues were immersed in fixative (4% paraformaldehyde in 0.1 M sodium phosphate buffer [pH 7.4]) overnight at 4°C. Mice were housed under standard conditions with ad libitum access to food and water. All animal procedures were approved by the Animal Care and Use Committee of Kawasaki Medical School (approval number: 20-013) and were conducted in accordance with institutional guidelines.

Human urinary bladder tissues were collected from 10 patients (seven males and three females) diagnosed with urinary bladder cancer who underwent radical cystectomy (Table 1). The urinary bladder tissues (1 cm^3^) with all layers were dissected from macroscopically benign lesions. Half of the tissue was further dissected to the urinary epithelial layer (Epi) and subepithelial layer (SubEpi). The tissues were immediately frozen in liquid nitrogen and stored at −80°C until use for glycolipid or RNA extraction. For histopathological analysis, a small portion of tissue with all layers was immersed in fixative (10% formaldehyde in 0.1 M sodium phosphate buffer [pH 7.4]) overnight at 4°C. Table 1 shows the clinicopathological features of the patients. Several patients underwent chemotherapy before radical cystectomy. Tumor staging was based on the Union for International Cancer Control TNM classification. The T category describes the primary tumor site and size, the N category describes regional lymph node involvement, and the M category describes the presence or otherwise of distant metastatic spread. T1 and T2 tumors are categorized as superficial, and T2, T3, and T4 tumors are invasive. The human study was conducted in compliance with the principles of the Declaration of Helsinki and approved by the Ethics Committee of Kawasaki Medical School (approval number: 3884), and written informed consent was obtained from all patients.

**Table 1:** Clinicopathological features of 10 cases with bladder tumor

Case no.	Age (year)	Sex	Histology	Grade	T	N	M	Stage	Treatment history before total cystectomy
1	73	Male	iUC with trophoblastic differentiation	High	3b	0	0	III	TUR-Bt
2	72	Male	iUC	High	3a	1	0	IV	TUR-Bt, GEM + CBDCA, BCG for 7 months
3	49	Male	iUC	High	2	0	0	II	TUR-Bt
4	69	Male	iUC	High	2	0	0	II	TUR-Bt, GEM + CDDP
5	79	Male	iUC with squamous differentiation	High	2	0	0	II	TUR-Bt
6	71	Male	iUC	High	2	0	0	II	TUR-Bt, BCG for 15 months
7	75	Male	iUC	High	2b	0	0	II	TUR-Bt
8	62	Female	iUC	High	4a	0	1b (ovary)	IV	TUR-Bt
9	75	Female	iUC with glandular differentiation	High	2	0	0	II	TUR-Bt
10	77	Female	iUC	High	4a	1	0	III	TUR-Bt, BCG for 76 months

CBDCA, carboplatin; CDDP, cisplatin; GEM, gemcitabine; iUC, invasive urothelial carcinoma; M, distant metastasis; N, lymph node involvement; T, tumor site and size; TUR-Bt, transurethral resection of the bladder tumor.

### GSL analysis by TLC

Sphingolipids were extracted from urinary bladder tissues of mice and humans as described previously (5). Briefly, 100 mg wet weight of mouse whole urinary bladder tissues pooled from four mice of each sex, or 100 mg wet weight of human urinary bladder tissues (total layer, Epi, and SubEpi) of case 4 (male), case 8 (female), and case 2 (male, treated by intravesical Bacille Calmette-Guerin (BCG) infusion, which depleted Epi before total cystectomy) were homogenized in four volumes of ice-cold distilled water in a Potter-Elvehjem glass homogenizer using a glass pestle. The 500 μl of each homogenate was combined with 4.5 ml chloroform/methanol (1:2, by volume) in a glass centrifuge tube and mixed thoroughly by vortexing. The sample tubes were left for 30 min at room temperature with occasional shaking and centrifuged at 1,500 *g* for 10 min. The supernatants were collected in fresh glass tubes and evaporated under nitrogen flow. The dried residues were suspended in 0.9 ml chloroform/methanol (1:2, by volume) and 0.1 ml 1 N NaOH and incubated at 37°C for 2 h. The saponified samples were neutralized with 6 μl acetic acid and evaporated under nitrogen flow. The samples were suspended in 6 ml chloroform/methanol (1:2, by volume) and purified using a reverse-phase column (Bond Elut C-18; 3 ml/500 mg; Agilent Technologies, Inc, CA). Lipid fractions equivalent to 30 mg wet weight tissues were spotted onto silica gel-coated high-performance TLC (HPTLC) plates (Merck, Darmstadt, Germany) and developed with a solvent system of chloroform/methanol/0.2% CaCl_2_ (60:35:8, by volume). GSLs were visualized using orcinol sulfate reagent. To distinguish GlcCer from galactosylceramide (GalCer), lipid fractions equivalent to 60 mg wet weight tissues were spotted onto borate-impregnated silica gel-coated HPTLC plates (17) and developed with a solvent system of chloroform/methanol/water/concentrated NH_4_OH (280:70:6:1, by volume). GSLs used as TLC standards were a neutral GSL mixture (Matreya LLC, PA), which contains GalCer, lactosylceramide (LacCer), globotriaosylceramide (Gb3Cer), and globotetraosylceramide (Gb4Cer), a TLC lactosylceramide and sialosyl derivative mixture, which contains LacCer, Neu5Ac-Neu5Ac-Gal-Glc-Ceramide (GD3), and GM3 (Matreya LLC), and Glucocerebrosides, buttermilk, which contains GlcCer (d18:1-22:0) (Matreya LLC).

### Structural analysis of GSLs by LC-MS

The structures of GSLs in mouse extracts from pooled four male whole urinary bladder tissues and in human extracts from Epi of case 4 (male) were characterized using an LC-MS instrument equipped with a quadrupole TOF MS (Shimadzu LCMS-9030, Shimadzu Corp, Kyoto, Japan) and LabSolutions, version 5.99 SP2 software as described previously (18). The LC conditions were solvent A, aqueous ammonia/acetic acid/distilled water/methanol/isopropanol (0.1:0.1:25:25:50, by volume); solvent B, aqueous ammonia/acetic acid/distilled water/methanol/isopropanol (0.1:0.1:2:48:50, by volume); the elution program was 0% solvent B in solvent A from 0 to 10 min, 0–100% solvent B from 10 to 35 min, 100% solvent B from 35 to 44 min, and 100–0% solvent B from 44 to 45 min, and 0% solvent B from 45 to 55 min; the flow rate was 50 μl/min; a Develosil C30 1 mm × 50 mm (Nomura Chemical, Nagoya, Japan) column was used; and the oven temperature was 40°C. The MS conditions were interface nebulizer gas flow rate 2.0 l/min, heating gas flow rate 10 l/min, interface temperature 200°C, drying gas flow rate 10 l/min, DL temperature 250°C, heat block temperature 400°C, and ESI source voltage –3.5 kV. For MS^2^ spectrum measurement in negative ion mode, scanning from *m/z* 50 to 2,000 was set to 0.1 s, the collision energy was 40 eV, and the detector voltage was 2.5 kV. The ceramide structures of GSLs were assigned with MS^2^ spectra using the fragment profiles listed in Table 2 (18, 19).

**Table 2:**
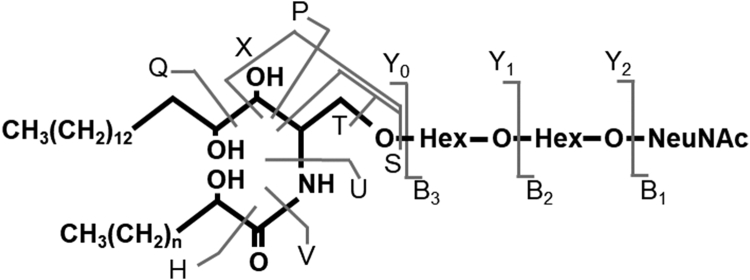
Fragmentation schema of GM3 containing 4-hydroxysphinganine and *m/z* values of reporter fragments of ceramide

	H	V	U	T	S	X
16:0	—	237	254	280	296	310
h16:0	225	253	271	296	312	326
18:0	—	265	282	308	324	338
h18:0	253	281	299	324	340	354
20:0	—	293	310	336	352	366
h20:0	281	309	327	352	368	382
22:0	—	321	338	364	380	394
h22:0	309	337	355	380	396	410
23:0	—	335	352	378	394	408
h23:0	323	351	369	394	410	424
24:1	—	347	364	390	406	420
h24:1	335	363	381	406	422	436
24:0	—	349	366	392	408	422
h24:0	337	365	383	408	424	438
26:1	—	375	392	418	434	448
h26:1	363	391	409	434	450	464
26:0	—	377	394	420	436	450
h26:0	365	393	411	436	452	466

	P	Q
d18:1	237	
t18:0	255	225
t20:0	283	253

### Multiple reaction monitoring analysis by LC-MS

The amounts of GSLs in whole urinary bladder tissues of four mice (two males and two females) and in Epi and SubEpi of human cases 2, 4, and 8 were determined independently via multiple reaction monitoring (MRM) using triple quadrupole LC-MS (Shimadzu LCMS-8060NX) and LabSolutions, version 5.113. Total lipids were extracted from tissue homogenate with 1 ml chloroform/methanol (1:2, by volume) containing 250 nM GlcCer (d18:1/16:0-d3), LacCer (d18:1/17:0), GM3 (d18:1/18:0-d3), and GD3 (d18:1/18:0-d3) as internal standards. After the removal of tissue residue by centrifugation, the supernatant was dried up under nitrogen flow. Total lipids were resuspended with 180 μl of chloroform/methanol (1:2, by volume) and 20 μl 1 N NaOH and incubated at 37°C for 2 h and then dried up under nitrogen flow. GSLs were purified using a reverse-phase column (MonoSpin C18 FF; GL Sciences). The LC conditions are described in “Structural analysis of GSLs by LC-MS” section. The MS conditions were interface nebulizer gas flow rate 2.0 l/min, heating gas flow rate 10 l/min, interface temperature 200°C, dissolved temperature 355°C, DL temperature 250°C, heat block temperature 300°C, drying gas flow rate 10 l/min, probe voltage 1.0 kV, and focus voltage 2.0 kV. MRM transitions and collision energy are shown in Supplemental Table S2 (20). Peak area of each GSLs was integrated using LabSolutions Insight, version 3.8 SP1, and the amount of GSLs is expressed as relative to internal standards indicated in Supplemental Table S2.

### Quantitative RT-PCR

Total RNA was extracted from mouse whole urinary bladder tissues (n = 4, two males and two females) and human urinary bladder tissues (total layer of case 1, and total, Epi, and SubEpi of cases 2, 4, 8, using the RNeasy Plus Universal Mini Kit [Qiagen, Hilden, Germany]) in accordance with the manufacturer’s protocol. Total RNA was quantified by NanoDrop One spectrophotometer (Thermo Fisher Scientific, MA). Human total RNA Master Panel II (catalog no.: 636643; Clontech, WI), and total RNA extracted from total layer of case 1 was used to examine differential expression levels among human tissues. Complementary DNA (cDNA) was synthesized from 1 μg total RNA using the PrimeScript RT Reagent Kit with gDNA Eraser (TaKaRa Bio, Kusatsu, Japan).

Quantitative RT-PCR (qRT-PCR) for *DES1*, *DES2*, *FA2H*, SPT LCB subunit 1 (*SPTLC1*), SPT LCB subunit 2 (*SPTLC2*), SPT LCB subunit 3 (*SPTLC3*), SPT small subunit A (*SPTSSA*), SPT small subunit B (*SPTSSB*), six different CER isozymes (*CERS1*–*6*), and *GAPDH* was performed using FastStart Universal Probe Master (Rox) and Universal ProbeLibrary (Roche, Basel, Switzerland) in a StepOnePlus™ Real-Time PCR System (Applied Biosystems/Thermo Fisher Scientific, MA). The primer sequences and probe numbers are listed in Supplemental Table S1. TaqMan Rodent GAPDH Control Reagents (Applied Biosystems/Thermo Fisher Scientific) were used to quantify *Gapdh* expression in mice. PCR conditions were 50°C for 2 min and 95°C for 10 min, followed by 40 cycles of denaturation at 95°C for 10 s, and annealing/extension at 60°C for 30 s. mRNA levels were calculated using the comparative threshold cycle method and normalized to that of *Gapdh*. Data in human cases were shown with technical triplicate.

### Histopathological analysis

Formaldehyde-fixed urinary bladder tissues of mice and humans were processed into paraffin-embedded blocks, sectioned, and stained with hematoxylin and eosin. For immunohistochemical studies, the slides were dewaxed and subjected to antigen retrieval by boiling for 10 min in 10 mM citric acid (pH 6.0). The slides were incubated with 1% bovine serum albumin/phosphate-buffered saline with 0.5% Triton X-100 for 1 h to block nonspecific binding and increase the penetration of antibodies. The slides were incubated with the primary antibodies overnight at 20°C followed by the secondary antibodies for 2 h at room temperature. The primary antibodies were rabbit polyclonal anti-human DES2 (dilution rate 1:50, catalog no.: PA5-24082; Thermo Fisher Scientific) and mouse monoclonal anti-mouse Uroplakin III, which is the marker of apical membrane of umbrella cell (dilution rate 1:50, catalog no.: ab78197; Abcam, Cambridge, UK) antibodies. The primary antibody was omitted as a negative control. The species-specific secondary antibodies conjugated to Alexa Fluor 488 or Alexa Fluor 546 (Thermo Fisher Scientific) were used at a dilution of 1:200. After nuclear staining with Hoechst 33342 (Dojindo, Tokyo, Japan), slides were mounted in Vectashield (Vector Laboratories, CA) and examined with a confocal laser scanning microscope (LSM700; Carl Zeiss, Oberkochen, Germany). Quantification of mean fluorescent intensity was done using ImageJ (National Institutes of Health) (21).

### Statistical analysis

Statistical analysis was performed using Student’s *t-*test in Prism, version 7.00 for Windows (GraphPad Software, CA). Statistical significance was defined as *P* < 0.05. Data are means ± SDs.

## Results

### GSLs in urinary bladder tissues of mice and humans

**Fig. 1:**
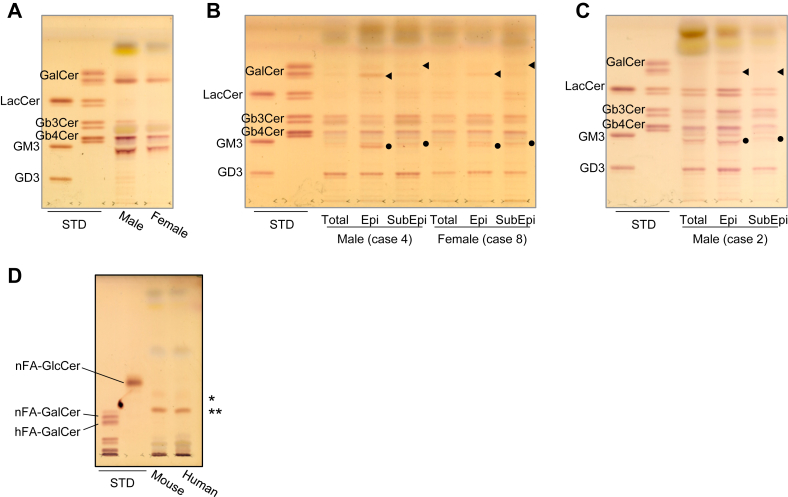
GSL analysis of urinary bladder tissues in mice and humans using TLC. Lipid fractions equivalent to 30 mg wet weight urinary bladder tissues of mice and human were spotted and developed with a solvent system of chloroform/methanol/0.2% CaCl_2_ (60:35:8, by volume). GSLs were visualized using orcinol sulfate reagent. ◄ and ⬤ denote HexCer and GM3, respectively. The GSLs in mouse urinary bladder tissues in male and female (A). The GSLs in human urinary bladder tissues in male (case 4) and female (case 8) (B). The GSLs in human urinary bladder tissues in case 2 (C). Lipid fractions equivalent to 60 mg wet weight urinary bladder tissues of mice and human (Epi of case 4) were spotted onto the borate-impregnated silica gel-coated HPTLC plate and developed with a solvent system of chloroform/methanol/water/concentrated NH_4_OH (280:70:6:1, by volume) to separate GlcCer and GalCer. (∗) and (∗∗) denote HexCer (D). STD, standard.

TLC indicated that in mouse urinary bladders the major GSLs were hexosylceramide (HexCer), Gb3Cer, Gb4Cer, and GM3 both in males and females (Fig. 1A). In human urinary bladders, the major GSLs were HexCer, LacCer, Gb3Cer, Gb4Cer, GM3, and GD3 both in male (case 4) and female (case 8) (Fig. 1B). In humans, bands in the HexCer and GM3 migrated slower in Epi than in SubEpi. In case 2, of which Epis were depleted by the intravesical BCG infusion before total cystectomy, the bands in the HexCer and GM3 migrated similarly in Epi and SubEpi (Fig. 1C). These findings suggest structural differences in GSL between Epi and SubEpi. It is possible that epithelial cells have a more hydrophilic structure, such as phytoceramide or 2-hydroxy fatty acid. By the TLC with the borate-impregnated silica gel-coated HPTLC plates, the minor upper bands of HexCer (∗) in the urinary bladder of both mice and human (Epi of case 4), migrated faster than the standard of GalCer, suggesting that these bands are GlcCer. On the other hand, the major band of HexCer (∗∗) in both mice and human migrated slightly faster but was not clearly separated from GalCer (Fig. 1D). Considering these results together with the results of normal-phase TLC, the major band of HexCer (∗∗) is likely GlcCer with phytoceramide.

### Structural characterization and quantification of GSLs in mouse urinary bladder tissue

Thirty-five GSL species were characterized by LC-MS in lipid extracts from whole urinary bladder tissues pooled from four male mice. Phytoceramide structures with 18- and 20-carbon LCBs and 2-hydroxy fatty acid were enriched in HexCer and GM3 but were not detected in LacCer, Gb3Cer, and Gb4Cer (Table 3).

**Table3:** Assigned GSL molecules of urinary bladder in mouse and human

A: Mouse urinary bladder					
	d18:0	d18:1	t18:0	d20:1	t20:0
HexCer		d18:1/16:0	t18:0/h16:0	d20:1/h24:0	t20:0/h24:0
		d18:1/24:1	t18:0/h22:0		t20:0/h24:1
		d18:1/24:0	t18:0/h23:0		t20:0/h26:0
		d18:1/h16:0	t18:0/h24:1		
		d18:1/h22:0	t18:0/h24:0		
		d18:1/h24:1			
		d18:1/h24:0			
LacCer		d18:1/16:0			
		d18:1/22:0			
		d18:1/24:1			
		d18:1/24:0			
Gb3Cer		d18:1/16:0			
		d18:1/20:0			
		d18:1/22:0			
		d18:1/24:1			
		d18:1/24:0			
Gb4Cer	d18:0/20:0	d18:1/16:0			
		d18:1/22:0			
		d18:1/23:0			
		d18:1/24:1			
		d18:1/24:0			
GM3		d18:1/16:0			t20:0/h26:0
		d18:1/22:0			t20:0/h24:0
		d18:1/24:1			
		d18:1/24:0			

B: Human urinary bladder			
	d18:1	t18:0	t20:0
HexCer	d18:1/16:0	t18:0/24:0	t20:0/24:0
	d18:1/22:0	t18:0/h16:0	t20:0/h24:1
	d18:1/24:1	t18:0/h20:0	t20:0/h24:0
	d18:1/24:0	t18:0/h22:0	
	d18:1/h16:0	t18:0/h23:0	
	d18:1/h22:0	t18:0/h24:1	
	d18:1/h23:0	t18:0/h24:0	
	d18:1/h24:1		
	d18:1/h24:0		
LacCer	d18:1/16:0		
	d18:1/24:1		
	d18:1/24:0		
Gb3Cer	d18:1/16:0		
	d18:1/22:0		
	d18:1/24:1		
	d18:1/24:0		
Gb4Cer	d18:1/16:0		
	d18:1/22:0		
	d18:1/24:1		
	d18:1/24:0		
GM3	d18:1/16:0	t18:0/h22:0	t20:0/h24:1
	d18:1/22:0	t18:0/h23:0	t20:0/h24:0
	d18:1/24:1	t18:0/h24:1	
	d18:1/24:0	t18:0/h24:0	
GD3	d18:1/24:1	t18:0/h24:1	t20:0/h24:0
	d18:1/24:0		

**Fig. 2:**
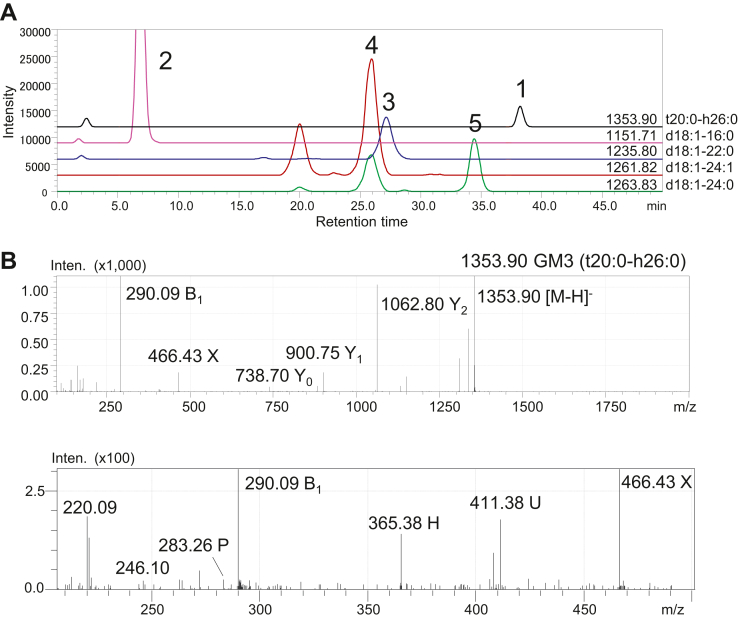
Structural analysis of GM3s in the mouse urinary bladder using LC-MS. A: Mass chromatograms of five GM3 molecules. B: MS^2^ spectra of peak 1 for *m/z* 1353.90 represented GM3 (t20:0-h26:0), which is characterized by reporter fragment ions of *m/z* 283.26 P and *m/z* 365.38 H. Four other peaks in (A) were confirmed to be GM3 (d18:1-16:0) for *m/z* 1151.71 peak 2, GM3 (d18:1-22:0) for *m/z* 1235.80 peak 3, GM3 (d18:1-24:1) for *m/z* 1261.82 peak 4, and GM3 (d18:1-24:0) for *m/z* 1263.83 peak 5.

Five GM3 species were detected (Table 3). Figure 2A shows the mass chromatograms of five peaks for GM3, which were confirmed by MS^2^ analysis to be GM3 (t20:0-h26:0) for *m/z* 1353.90 peak 1, GM3 (d18:1-16:0) for *m/z* 1151.71 peak 2, GM3 (d18:1-22:0) for *m/z* 1235.80 peak 3, GM3 (d18:1-24:1) for *m/z* 1261.82 peak 4, and GM3 (d18:1-24:0) for *m/z* 1263.83 peak 5. The MS^2^ spectrum of peak 1 identified GM3 with a 20-carbon phyto-type LCB (4-hydroxyeicosasphinganine, t20:0) and 2-hydroxyhexacosanoic acid (h26:0), which are characterized by reporter fragment ions of *m/z* 283.26 P and *m/z* 365.38 H (Fig. 2B). H and P ions were reporter fragments for 2-hydroxy fatty acid and LCB, respectively (Table 2) (19).

**Fig. 3:**
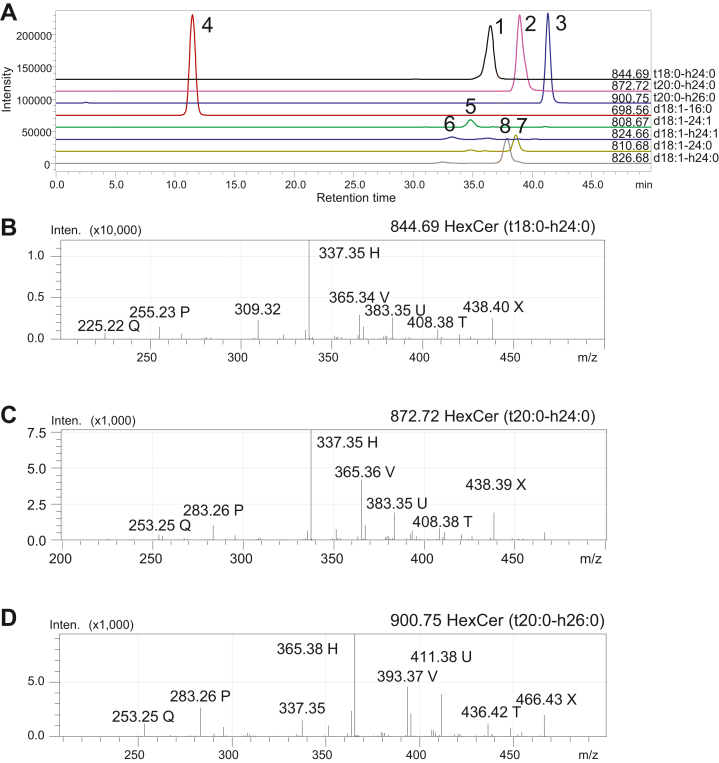
Structural analysis of HexCers in the mouse urinary bladder using LC-MS. A: Mass chromatograms of eight HexCer molecules. B: MS^2^ spectrum of peak 1 for *m/z* 844.69 represents HexCer (t18:0-h24:0), which is characterized by reporter fragment ions of *m/z* 255.23 P and *m/z* 337.35 H. C: MS^2^ spectrum of peak 2 for *m/z* 872.72 represents HexCer (t20:0-h24:0), which is characterized by reporter fragment ions of *m/z* 283.26 P and *m/z* 337.35 H. D: MS^2^ spectrum of peak 3 for *m/z* 900.75 represents HexCer (t20:0-h26:0), which is characterized by reporter fragment ions of *m/z* 283.26 P and *m/z* 365.38 H. Peaks 4 to 8 in (A) represent HexCer (d18:1–16:0) for *m/z* 698.56, HexCer (d18:1-24:1) for *m/z* 808.67, HexCer (d18:1-h24:1) for *m/z* 824.66, HexCer (d18:1-24:0) for *m/z* 810.68, and HexCer (d18:1-h24:0) for *m/z* 826.68, respectively, by MS^2^ analysis (data not shown).

Sixteen HexCer species were detected (Table 3). Figure 3A shows the mass chromatograms of eight peaks for HexCer, which were confirmed by MS^2^ analysis to be HexCer (t18:0-h24:0) for *m/z* 844.69 peak 1, HexCer (t20:0-h24:0) for *m/z* 872.72 peak 2, HexCer (t20:0-h26:0) for *m/z* 900.75 peak 3, HexCer (d18:1-16:0) for *m/z* 698.56 peak 4, HexCer (d18:1-24:1) for *m/z* 808.67 peak 5, HexCer (d18:1-h24:1) for *m/z* 824.66 peak 6, HexCer (d18:1-24:0) for *m/z* 810.68 peak 7, and HexCer (d18:1-h24:0) for *m/z* 826.68 peak 8. The MS^2^ spectrum of peak 1 for *m/z* 844.69 indicated HexCer with an 18-carbon phyto-type LCB and 2-hydroxy tetracosanoic acid (t18:0-h24:0), which are characterized by reporter fragment ions of *m/z* 255.23 P and *m/z* 337.35 H for 4-hydroxysphinganine (t18:0) carrying h24:0 fatty acid (Fig. 3B). The MS^2^ spectrum of peak 2 for *m/z* 872.72 indicated HexCer with a 20-carbon phyto-type LCB and 2-hydroxytetracosanoic acid (t20:0/h24:0), which are characterized by reporter fragment ions of *m/z* 283.26 P and *m/z* 337.35 H (Fig. 3C). The MS^2^ spectrum of peak 3 for *m/z* 900.75 indicated HexCer with a 20-carbon phyto-type LCB and 2-hydroxyhexacosanoic acid (t20:0-h26:0), which are characterized by reporter fragment ions of *m/z* 283.26 P and *m/z* 365.38 H (Fig. 3D).

**Fig. 4:**
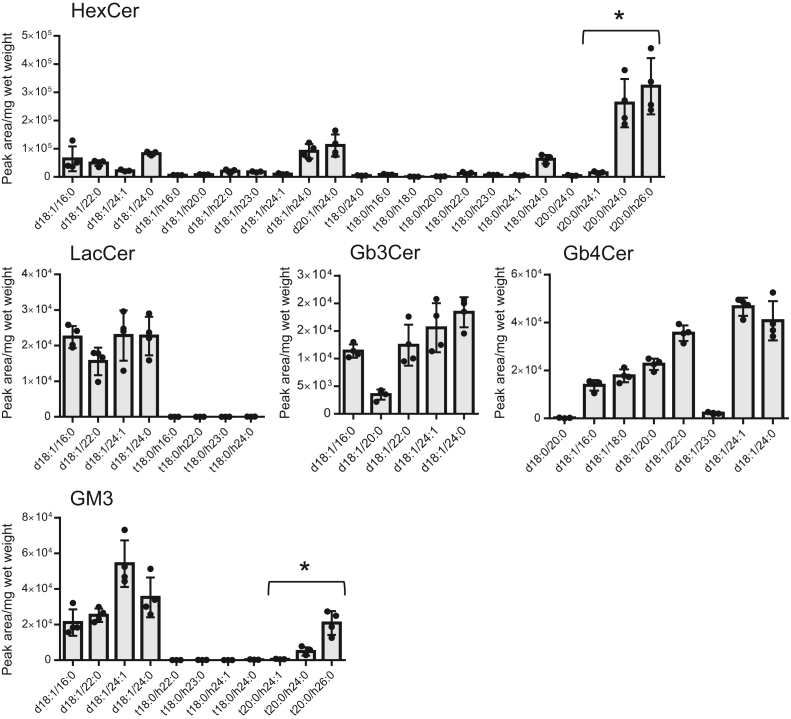
Amounts of assigned molecular species in GSLs in the mouse urinary bladder. The amounts of assigned molecular species in HexCer, LacCer, Gb3Cer, Cb4Cer, and GM3 in the mouse urinary bladder tissues were determined via MRM analysis as shown in supplemental Table S2. Phytoceramide structures with 20-carbon LCBs and 2-hydroxy fatty acids denoted by ∗ are present in significant amount of HexCer and GM3. Data are shown by means ± SDs (*n* = 4).

MRM analyses revealed that the amounts of phytoceramide structures with a 20-carbon LCB and with 2-hydroxy fatty acid (t20:0-h24:0 and t20:0-h26:0) were abundant in HexCer and GM3 but not in LacCer, Gb3Cer, and Gb4Cer (Fig. 4). Considering these results together with the results of TLC using borate-impregnated HPTLC plates (Fig. 1D), it can be concluded that the major HexCer molecular species in the mouse urinary bladder is GlcCer with phytoceramide structure not GalCer with ceramide structure.

### Structural characterization and quantification of GSL in human urinary bladder Epi

Forty GSL species were detected by LC-MS in Epi of case 4. Phytoceramide structures with 18- and 20-carbon LCBs and 2-hydroxy fatty acid were enriched in HexCer, GM3, and GD3 but were not detected in LacCer, Gb3Cer, and Gb4Cer (Table 3).

**Fig. 5:**
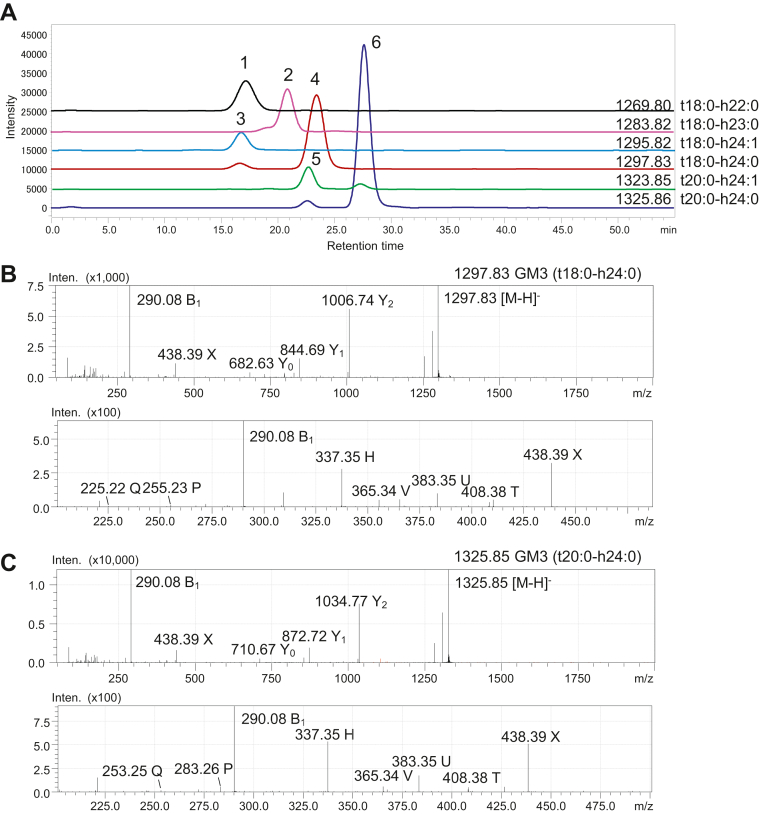
Structural analysis of GM3s containing 4-hydroxysphinganine in the human urinary bladder using LC-MS. A: Mass chromatograms of six GM3 molecules. B: MS^2^ spectrum of peak 4 for *m/z* 1297.83 represents GM3 (t18:0-h24:0), which is characterized by reporter fragment ions of *m/z* 255.23 P and *m/z* 337.35 H. C: MS^2^ spectrum of peak 6 for *m/z* 1325.86 represents GM3 (t20:0-h24:0), which is characterized by reporter fragment ions of *m/z* 283.26 P and *m/z* 337.35 H. Peaks 1, 2, 3, and 5 in (A) are confirmed as GM3 (t18:0-h22:0) for *m/z* 1269.80, GM3 (t18:0-h23:0) for *m/z* 1283.82, GM3 (t18:0-h24:1) for *m/z* 1295.82, and GM3 (t20:0-h24:1) for *m/z* 1323.85, respectively, by MS^2^ analysis (data not shown).

Ten GM3 species were detected (Table 3). Figure 5A shows the mass chromatograms of six peaks for GM3 containing a phyto-type LCB, which were confirmed by MS^2^ analysis to be GM3 (t18:0-h22:0) for *m/z* 1269.80 peak 1, GM3 (t18:0-h23:0) for *m/z* 1283.872 peak 2, GM3 (t18:0-h24:1) for *m/z* 1295.82 peak3, GM3 (t18:0-h24:0) for *m/z* 1297.83 peak 4, GM3 (t20:0-h24:1) for *m/z* 1323.85 peak 5, and GM3 (t20:0-h24:0) for *m/z* 1325.86 peak 6. The MS^2^ spectrum of peak 4 for *m/z* 1297.83 indicated GM3 with an 18-carbon phyto-type LCB and 2-hydroxytetracosanoic acid (t18:0-h24:0), which are characterized by reporter fragment ions of *m/z* 255.23 P and *m/z* 337.35 H (Fig. 5B). The MS^2^ spectrum of peak 6 for *m/z* 1325.87 indicated GM3 with a 20-carbon phyto-type LCB and 2-hydroxytetracosanoic acid (t20:0-h24:0), which are characterized by reporter fragment ions of *m/z* 283.26 P and *m/z* 337.35 H (Fig. 5C).

Four GD3 species were detected (Table 3). Supplemental Fig. S1A shows the mass chromatograms of two peaks for GD3, which were confirmed by MS^2^ spectra to be GD3 (d18:1-24:1) for *m/z* 775.95 peak 1 and GD3 (t18:0-24:1) for *m/z* 784.95 peak 2. The molecular ions of GD3 were double charged [M-2H]^2−^. The MS^2^ spectrum of peak 1 for *m/z* 775.95 indicated GD3 (d18:1-24:1), which are characterized by fragment ions from ceramide of *m/z* 237.22 P, *m/z* 347.33 V, *m/z* 365.45 U, *m/z* 390.37 T, and *m/z* 406.36 S (Supplemental Fig. S1B). The MS^2^ spectrum of peak 2 for *m/z* 784.95 indicated GD3 (t18:0-24:1), which were characterized by fragment ions from ceramide of *m/z* 347.33 V, *m/z* 364.34 U, *m/z* 390.37 T, and *m/z* 420.38 X (Supplemental Fig. S1C).

**Fig. 6:**
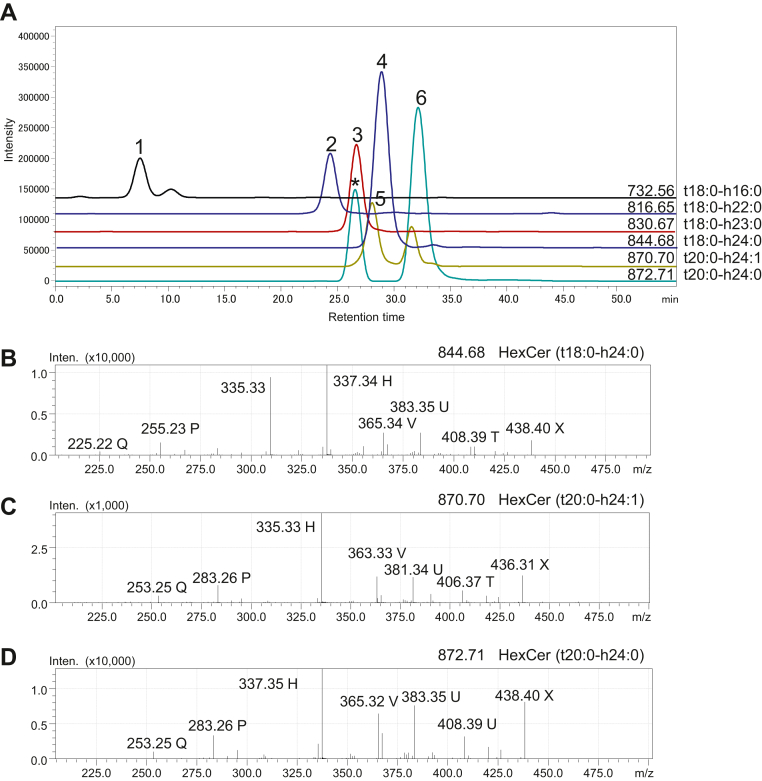
Structural analysis of HexCers containing 4-hydroxysphinganine in the human urinary bladder using LC-MS. A: Mass chromatograms of six HexCer molecules. B: MS^2^ spectrum of peak 4 for *m/z* 844.68 represents HexCer (t18:0-h24:0), which is characterized by reporter fragment ions of *m/z* 255.23 P and *m/z* 337.34 H. C: MS^2^ spectrum of peak 5 for *m/z* 870.70 represents HexCer (t20:0-h24:1), which is characterized by reporter fragment ions of *m/z* 283.26 P and *m/z* 335.33 H. D: MS^2^ spectrum of peak 6 for *m/z* 872.71 represents HexCer (t20:0-h24:0), which is characterized by reporter fragment ions of *m/z* 283.26 P and *m/z* 337.35 H. Peak marked with ∗ provides a fragment ion at *m/z* 168.04, demonstrating that the molecule is not a GSL but sphingomyelin.

Nineteen HexCer species were detected (Table 3). Figure 6A shows the mass chromatograms of six peaks for HexCer containing a phyto-type LCB, which were confirmed by MS^2^ analysis to be HexCer (t18:0-h16:0) for *m/z* 732.56 peak 1, HexCer (t18:0-h22:0) for *m/z* 816.65 peak 2, HexCer (t18:0-h23:0) for *m/z* 830.67 peak 3, HexCer (t18:0-h24:0) for *m/z* 844.68 peak 4, HexCer (t20:0-h24:1) for *m/z* 870.70 peak 5, and HexCer (t20:0-h24:0) for *m/z* 872.71 peak 6. The MS^2^ spectrum of peak 4 for *m/z* 844.68 indicated HexCer with an 18-carbon phyto-type LCB and 2-hydroxytetracosanoic acid (t18:0-h24:0), which are characterized by reporter fragment ions of *m/z* 255.23 P and *m/z* 337.34 H (Fig. 6B). The MS^2^ spectrum of peak 5 for *m/z* 870.70 indicated HexCer with a 20-carbon phyto-type LCB and 2-hydroxytetracosenoic acid (t20:0-h24:1), which were characterized by reporter fragment ions of *m/z* 283.26 P and *m/z* 335.33 H for a 20-carbon phyto-type LCB (t20:0) carrying h24:1 fatty acid (Fig. 6C). The MS^2^ spectrum of peak 6 for *m/z* 872.71 indicated HexCer with a 20-carbon phyto-type LCB and 2-hydroxytetracosanoic acid (t20:0-h24:0), which were characterized by reporter fragment ions of *m/z* 283.26 P and *m/z* 337.35 H (Fig. 6D).

**Fig. 7:**
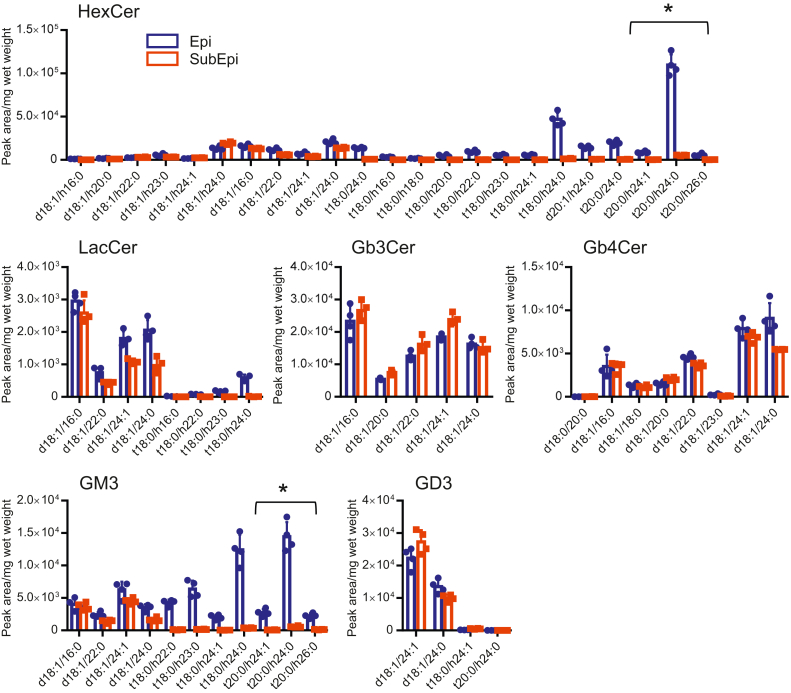
Amounts of assigned molecular species in GSLs in the Epi of human urinary bladder (case 4). The amounts of assigned molecular species in HexCer, LacCer, Gb3Cer, Cb4Cer, GM3, and GD3 in the Epi of human urinary bladder tissue (case 4) were determined via MRM analysis as shown in Supplemental Table S2. Phytoceramide structures with 20-carbon LCBs and 2-hydroxy fatty acids denoted by ∗ are present in significant amount of HexCer and GM3. Data are shown by mean ± SD with technical triplicate.

MRM analyses revealed that the amounts of phytoceramide structures with a 20-carbon LCB and with 2-hydroxy fatty acid (t20:0-h24:0) were abundant in HexCer and GM3 in case 4 (Fig. 7) and case 8 (Supplemental Fig. S2), but not in case 2, of which Epis were depleted by intravesical BCG infusion before total cystectomy (Supplemental Fig. S3). These findings indicate that phytoceramide structures with a 20-carbon LCB and 2-hydroxy fatty acids are abundant in particular GSL species, HexCer and GM3 in urinary epithelial cells in human. Considering these results together with the results of TLC using borate-impregnated HPTLC plates (Fig. 1D), it can be concluded that the major HexCer molecular species in the human urinary bladder is also GlcCer with phytoceramide structure and not GalCer with ceramide structure.

### *Des1*, *Des2*, *Fa2h*, *Sptlc1-3*, *Sptssa*, *Sptssb*, and *Cers1-6* expressions in mouse urinary bladder tissues

**Fig. 8:**
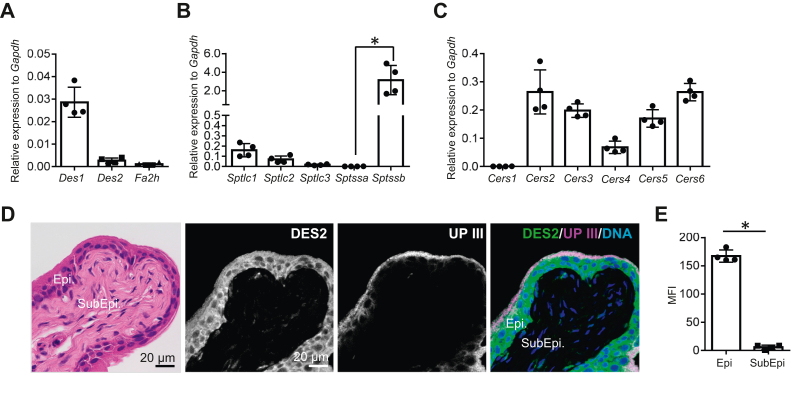
Expressions of *Des1*, *Des2*, *Fa2h*, *Sptlc1*-*3*, *Sptssa*, *Sptlcb*, *and Cers1*-*6* in mouse whole urinary bladders. The expression of *Des1*, *Des2*, and *Fa2h* (A), *Sptlc1∼3*, *Sptssa*, and *Sptssb* (B), *Cers1∼6* (C) in the whole urinary bladders of mice by qPCR. The expression of DES2 in the mouse urinary bladder tissues via immunohistochemistry (D). Quantification of DES2 immunofluorescence between Epi and SubEpi (E). Data are shown by means ± SDs (*n* = 4). ∗*P* < 0.05. STD, standard; UP III, uroplakin III, DNA, nuclear staining.

The expressions of *Des1*, *Des2*, *Fa2h*, *Sptlc1-3*, *Sptssa*, *Sptssb*, and *Cers1-6* were analyzed by qPCR in mouse whole urinary bladder tissues (*n* = 4). The expressions of *Des2* and *Fa2h* were lower than *Des1* but detected in significant amount (Fig. 8A). The expressions of *Sptlc1*, *Sptlc2*, *and Sptlc3* were detected in this order, and the expression of *Sptssb*, which are responsible for the generation of 20-carbon LCBs, was significantly higher than *Sptssa* and highest among tissues analyzed (Fig. 8B). Among the expressions of *Cers1-6*, the expressions of *Cers2*, *Cers3*, *Cers5*, *and Cers6* were dominant (Fig. 8C). Immunohistochemical analysis of mouse urinary bladder tissues showed that the expression of DES2 was found exclusively in urinary epithelial cells (Fig. 8D, E). These findings support that GSLs containing phytoceramide structures with 20-carbon LCBs and 2-hydroxy fatty acid are abundant in mouse urinary epithelial cells.

### *DES1*, *DES2*, *FA2H*, *SPTLC1-3*, *SPTSSA*, *SPTSSB*, and *CERS1-6* expression in human urinary bladder tissues

**Fig. 9:**
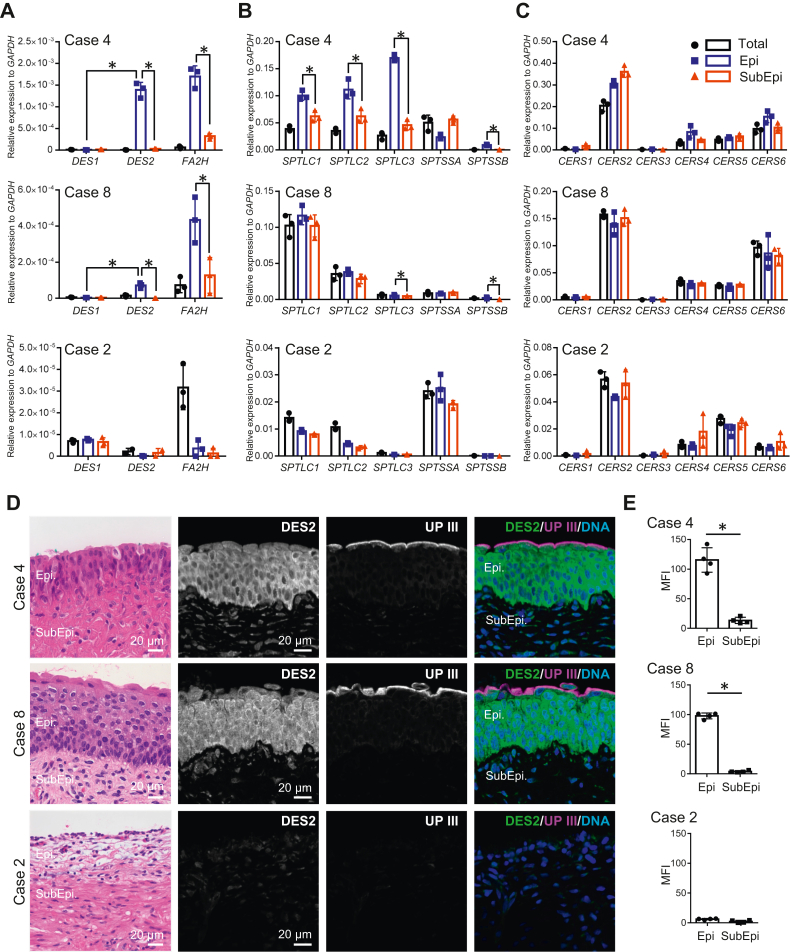
Expressions of *DES1*, *DES2*, *FA2H*, *SPTLC1*-*3*, *SPTSSA*, *SPTSSB*, and *CERS1*-*6* in human urinary bladder tissues. The expression of *DES1*, *DES2*, and *FA2H* (A), *SPTLC1*-*3*, *SPTSSA*, and *SPTSSB* (B), and *CERS1*-*6* (C) in the total layer, Epi, and SubEpi in human urinary bladders in case 4, case 8, and case 2 by qPCR. The expression of DES2 in the human urinary bladder tissues in case 4, case 8, and case 2 by immunohistochemical study (D). Quantification of DES2 immunofluorescence between Epi and SubEpi in case 4, case 8, and case 2 (E). Data are shown by means ± SDs by technical triplicate. ∗*P* < 0.05. MFI, mean fluorescent intensity; UP III, uroplakin III, DNA, nuclear staining.

The expressions of *DES1*, *DES2*, *FA2H*, *SPTLC1*-*3*, *SPTSSA*, *SPTSSB*, and *CERS1-6* were analyzed by qPCR in human urinary bladder tissues (total layer, Epi, and SubEpi) from case 4 (male), case 8 (female,), and case 2 (female, treated by intravesical BCG infusion, which depleted Epi before total cystectomy). In case 4 and 8, the expressions of *DES2* and *FA2H* in Epi were significantly higher than SubEpi (Fig. 9A). The expressions of *SPTLC1*-*3* were detected and higher in Epi than in SubEpi (Fig. 9B). The expression of *SPTSSB* was significantly higher in Epi than SubEpi. Among the expressions of *CERS1-6*, the expression of *CERS2* was dominant (Fig. 9C). In case 2, most of these expressions were decreased compared with cases 4 and 8, and the abundance of *DES2*, *FA2H*, *SPTLC3*, and *SPTSSB* expressions in Epi was not observed (Fig. 9A–C). Immunohistochemical analysis of human urinary bladder tissues showed that the expression of DES2 was found in urinary epithelial cells in both cases 4 and 8, but not in case 2, where negative staining for uroplakin III demonstrated epithelial cell depletion (Fig. 9D, E). These findings confirm that GSLs containing phytoceramides with 20-carbon LCBs and 2-hydroxy fatty acids are abundant in human urinary epithelial cells.

### *DES1*, *DES2*, *FA2H*, *SPTLC1*-*3*, *SPTSSA*, and *SPTSSB* expression in human tissues

**Fig. 10:**
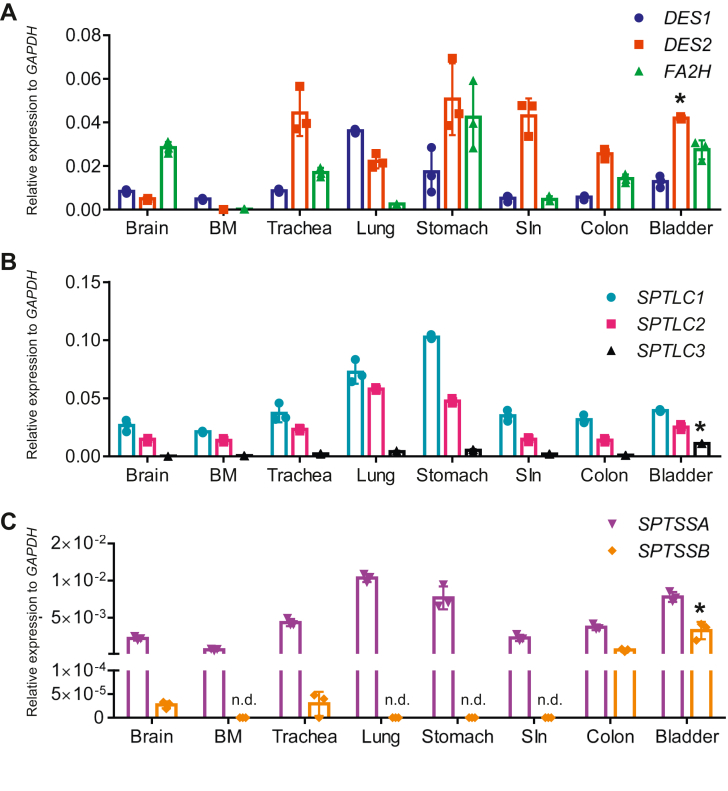
Expressions of *DES1*, *DES2*, *FA2H*, *SPTLC1*, *SPTLC2*, *SPTCL3*, *SPTSSA*, and *SPTSSB* in a human cDNA library. The expressions of *DES1*, *DES2*, and *FA2H* (A), *SPTLC1∼3* (B), *SPTSSA* and *SPTSSB* (C) by qPCR in a human cDNA library (Human total RNA Master Panel II, Clontech, WI) and cDNA synthesized from RNA extracted from total layer of case 1. Data are shown by means ± SDs with technical triplicate. ∗*P* < 0.05. n.d., not detected.

The expressions of *DES1*, *DES2*, *FA2H*, *SPTLC1*-*3*, *SPTSSA*, and *SPTSSB* in the human tissues were analyzed by qPCR using the human cDNA library and cDNA synthesized from RNA extracted from total layer of urinary bladder of case 1 (Fig. 10). The expression of *DES2* in urinary bladder was higher than that in brain and similar to gastrointestinal tract. The expression of *FA2H* in urinary bladder was similar to brain. The expressions of *SPTLC3* and *SPTSSB* in urinary bladder were higher than brain. The significant expression of *SPTSSB* was also detected in colon. These findings support that GSLs containing phytoceramides with a 20-carbon LCB are produced predominantly in human urinary bladder.

## Discussion

GSLs are composed of a polar head glycan chain and a hydrophobic tail ceramide (1, 2, 3). The structure of ceramide varies among tissues in terms of the carbon chain length, degree of desaturation, and hydroxylation in the *N*-acyl chain and LCB. However, the biological roles of these variabilities are unclear. Six distinct mammalian CERs (CERS1-6) that have preferences for fatty acyl-CoAs of different carbon chain lengths as substrates produce variation in the *N*-acyl carbon chain length (6, 7). CERS1-6 produce 2-hydroxy fatty acids containing ceramide as same as nonhydroxy fatty acids containing ceramide (22). Mutations in *CERS1* cause autosomal recessive progressive myoclonic epilepsy (23). Mutations in *CERS3* cause autosomal recessive congenital ichthyosis (24, 25). Fatty acid 2-hydroyxlase, encoded by *FA2H*, produces 2-hydroxy fatty acids containing sphingolipids in myelin (8, 9). Indeed, mutations in *FA2H* cause autosomal recessive leukodystrophy with spastic paraplegia in humans and mice (26, 27). Structural variation in LCBs is produced by mammalian SPTs, which are heterotrimeric proteins consisting of a large subunit dimer (SPTLC1 and SPTLC2 or SPTLC3) and one of two small subunits (SPTSSA or SPTSSB). The SPTLC1, SPTLC2, and SPTSSA complex condenses a palmitoyl-CoA and serine to generate an 18-carbon LCB, the most abundant LCB in mammalian cells. By contrast, the SPTLC1, SPTLC2, and SPTSSB complex favors stearoyl-CoA as a substrate, leading to a 20-carbon LCB, and that of SPTLC1, SPTLC3, and SPTSSB favors myristoyl-CoA or stearoyl-CoA, leading to a 16- or 20-carbon LCB, respectively (10, 28). Mutations in *SPTLC1* and *SPTLC2* cause autosomal dominant hereditary sensory neuropathy (29, 30). Twenty-carbon LCBs are present in gangliosides in human and mouse brains (31). In mice, a gain-of-function mutation in *Sptssb* enhances production of 20-carbon LCBs and results in profound neuropathological changes in the brain and retina, suggesting the importance of 20-carbon LCBs in vivo (32). DES1 produces ceramide with sphingenine, which is the most abundant LCB in mammalian cells. Mutations in *DES1* cause autosomal recessive hypomyelinating leukodystrophy in humans (33). DES2 produces phytoceramide with an additional hydroxy group at sphinganine C-4 (11). Phytoceramide structures are known to be abundant in mammalian small intestinal and renal epithelial cells (12). Although, their distinct functions are still unknown, because of the absence of human diseases or animal models for DES2 deficiency, their important role in the various epithelial cells is presumed.

In this study, we hypothesized that urothelial cells have unique GSL compositions to enable their stretching-and-shrinking membrane properties and examined the structural variation in the GSLs in the urinary bladders of mice and humans. Few studies have focused on membrane lipid GSLs in urinary bladder tumor or cell lines. Superficial urinary bladder tumors in humans show massive accumulation of GM3 (34). Human urothelial cell lines have HexCer, LacCer, Gb3Cer, Gb4Cer, Gal-GlcNAc-Gal-Glc-Ceramide, GM3, GalNAc-(Neu5Ac-)Gal-Glc-Ceramide (GM2), sialyl Gal-GlcNAc-Gal-Glc-Ceramide, and Neu5Ac-Gal-GalNAc-(Neu5Ac-)Gal-Glc-Ceramide. The major ceramide structures are composed of 16:0 and 24:0 fatty acids, and some of the HexCer and gangliosides contain more complex unsaturated and 2-hydroxy fatty acids of h16:0 and h24:0 (35). We found that phytoceramide structures with a 20-carbon LCB and 2-hydroxy fatty acid are enriched in HexCer and GM3 in the urinary bladder tissues of mouse and urinary Epis of human by TLC and LC-MS analysis (Figs. 4 and 7). These findings are supported by the gene expression of key enzymes involved in the synthesis of phytoceramides with a 20-carbon LCB and 2-hydroxy fatty acids (*DES2*, *FA2H*, *SPTLC3*, and *SPTSSB*) in the Epis (Figs. 8 and 9) (5, 26, 27). In addition, the immunohistochemical studies showed that DES2 was expressed exclusively in urothelial cells in both mouse and human.

The inner surface of the urinary bladder is lined by urothelial cells (16). The apical surface of urothelial cells is composed of umbrella cells, which dynamically change their morphology according to urine volume from cuboidal to highly stretched. The 70–90% of apical membrane of umbrella cells is covered by transmembrane protein called uroplakin (16, 36). Uroplakin forms highly specialized hexagonally packed protein plaques of 16 nm. Uroplakin plaque is thought to contribute to the barrier function and the stretching and shrinking of the apical membrane through the endocytosis and exocytosis of uroplakin-containing membrane.

Lipid rafts are cholesterol- and sphingolipid-enriched microdomains in the plasma membrane (37). The sphingolipids in the lipid rafts are known to be enriched in ceramide structures containing 2-hyroxy fatty acids. *FA2H*-knockdown in 3T3-L1 adipocytes increased the membrane mobility of raft-associated lipids and decreased transporter 4 and lipogenesis (38). Phase transition temperature of sphingomyelin composed of a hydroxy LCB and 2-hydroxy fatty acids was higher than that composed of a nonhydroxy LCB and fatty acids (39, 40). These findings suggest that the hydroxylation of LCB or fatty acid in ceramide structure plays important role for stabilization of lipid raft. The increase of chain length in LCB from 18-carbon to 20-carbon may decrease the membrane fluidity. It is speculated that the enrichment of phytoceramide structures with a 20-carbon LCB and 2-hydroxy fatty acids reduces membrane fluidity and allows tight plaque formation of uroplakin in the apical membrane of umbrella cells.

In conclusion, GSLs in urothelial cells of mouse and human urinary bladder are enriched in phytoceramide structures with a 20-carbon LCB and 2-hydroxy fatty acids in GlcCer and GM3. Our findings implicate that these unique ceramide structures play important role in stretching-and-shrinking membrane properties of transitional urothelial cells. Further research to address the biological significance and the pathophysiological contribution of these structures is warranted.

## Data Availability

The data generated or analyzed during this study are included in this published article (and its supplemental data files) or are available from the corresponding author upon reasonable request.

## Supplemental Data

This article contains supplemental data.

## Conflict of Interest

The authors declare that they have no conflicts of interest with the contents of this article.
