# Supplementary figures and images for: The Urinary Bladder is Rich in Glycosphingolipids Composed of Phytoceramides
Source: J Lipid Res. 2022 Oct 27;63(12):100303. doi: 10.1016/j.jlr.2022.100303 (PMC9708920; doi:10.1016/j.jlr.2022.100303)

# Supplementary Figure 1

**A**

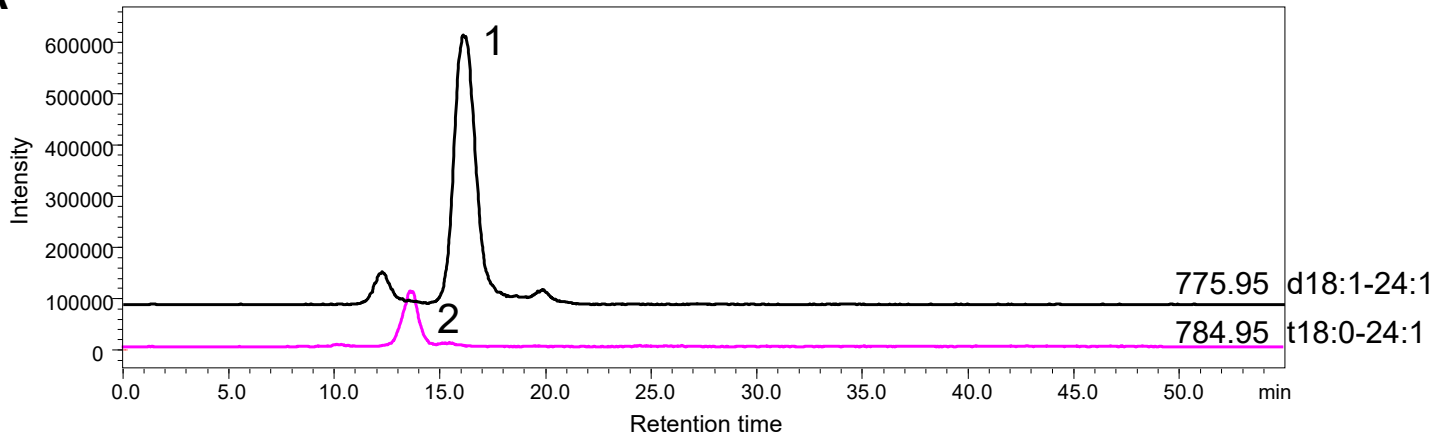

**B**

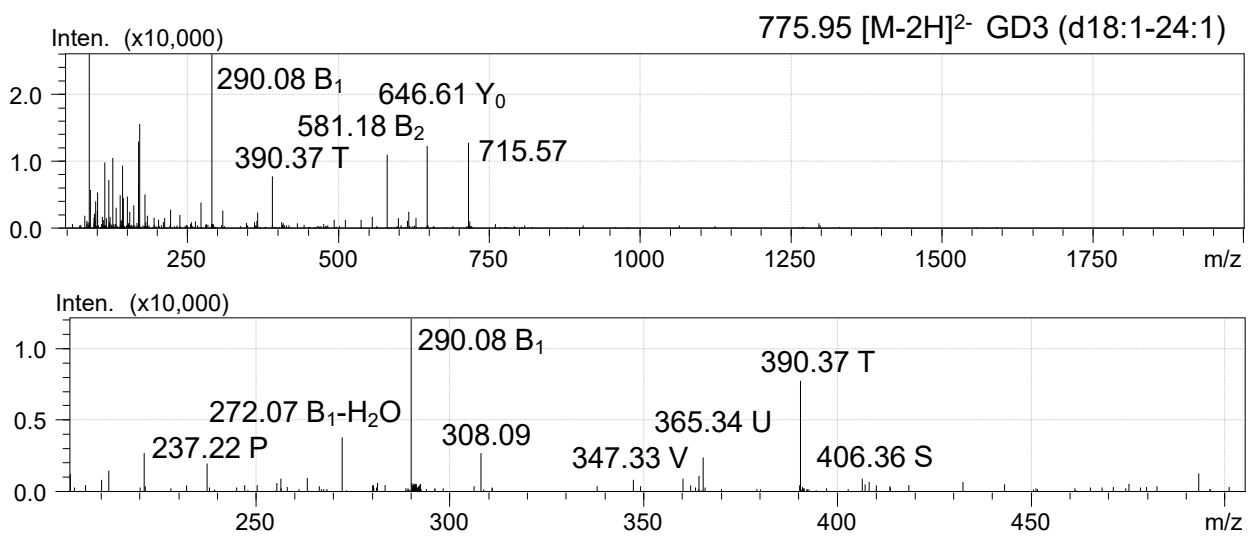

**C**

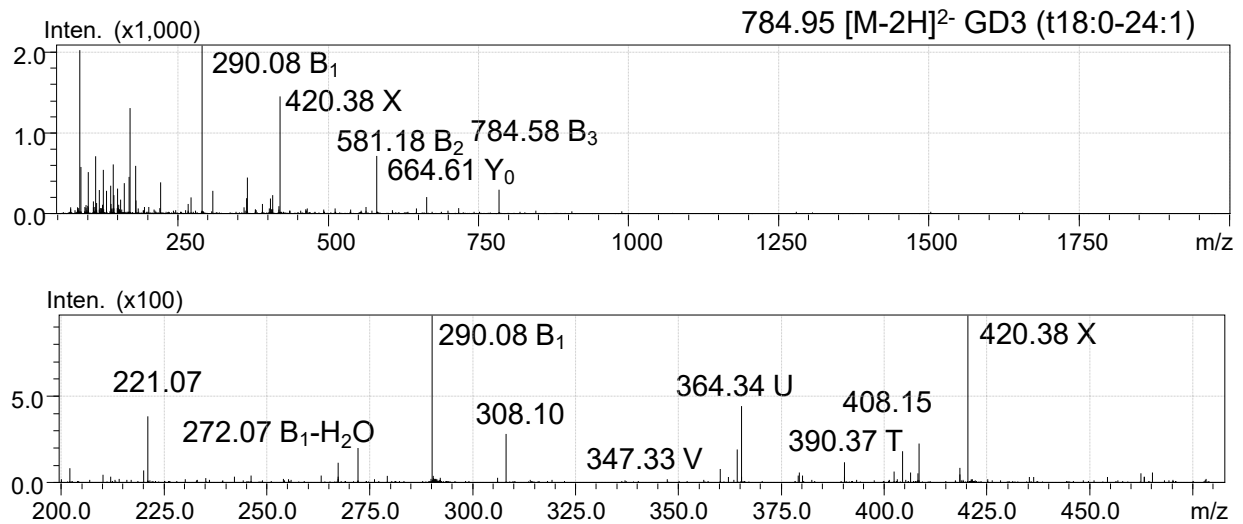

Supplementary Figure 2

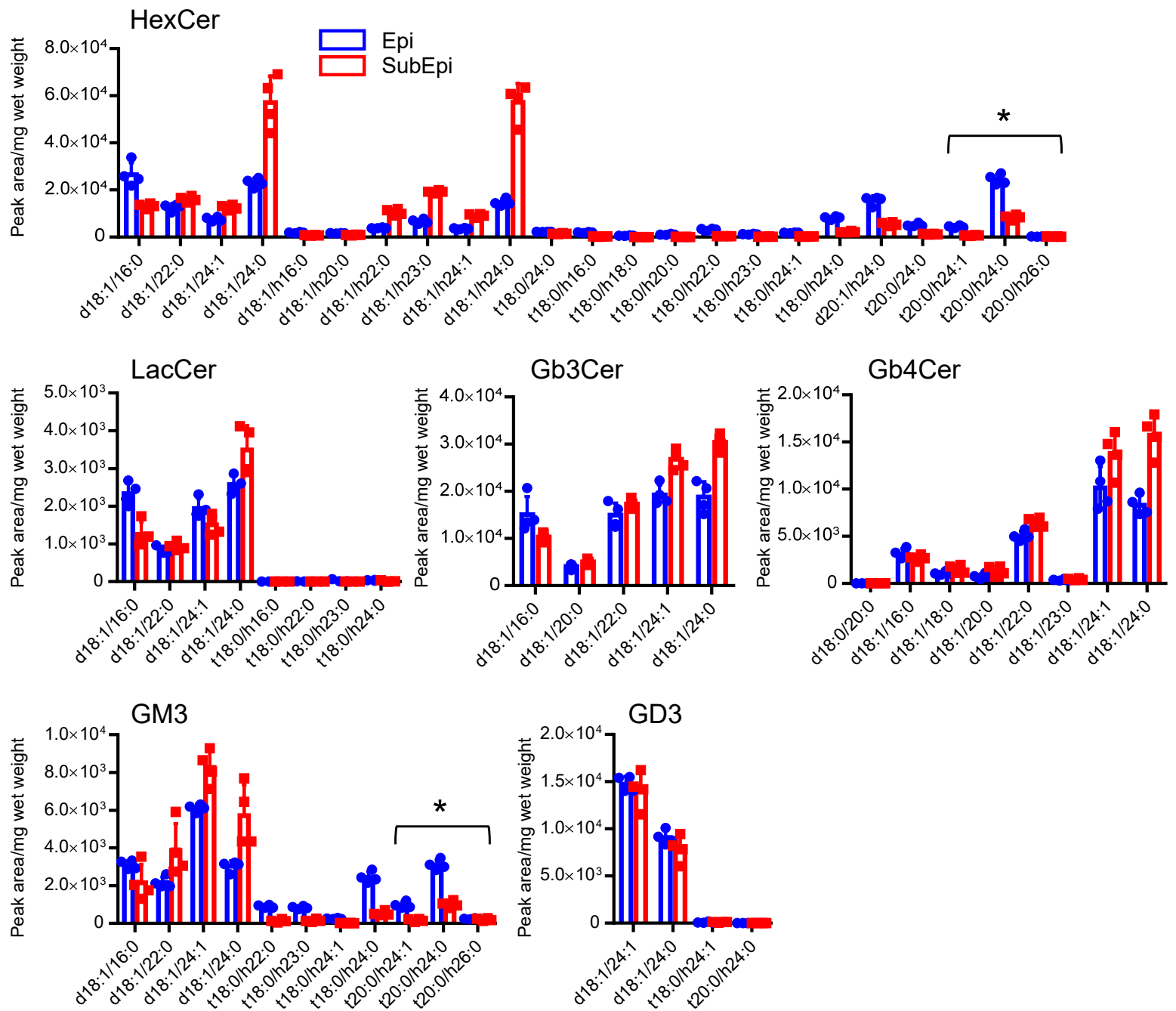

Supplementary Figure 3

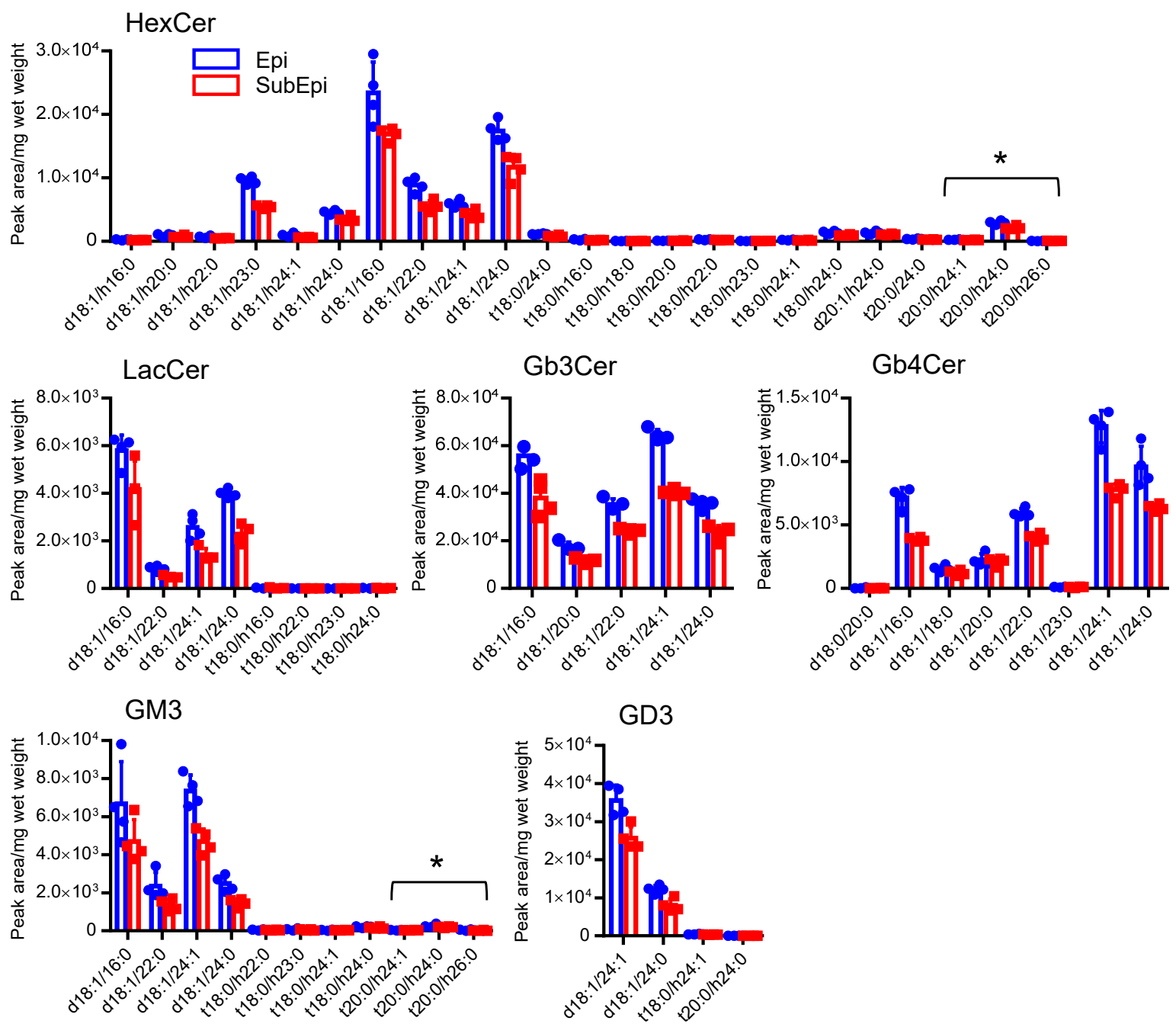

Supplement: Supplemental figures [file mmc1.pdf]
