# Supplementary material for: The Urinary Bladder is Rich in Glycosphingolipids Composed of Phytoceramides
Source: J Lipid Res. 2022 Oct 27;63(12):100303. doi: 10.1016/j.jlr.2022.100303 (PMC9708920; doi:10.1016/j.jlr.2022.100303)
Supplement: Supplemental tables [file mmc2.docx]

**Supplementary Table 1**

List of qPCR Primers and Probes

(A) Mouse

| **Gene Name** | **Accession No.** |  | **Sequence (5'-3')** | **Position** | **Probe No.** |
| --- | --- | --- | --- | --- | --- |
| *Des1* | NM_007853.5 | F | GGCTATCATAACGAGCACCAT | 867-887 | 60 |
|  |  | R | CACTTGCGATCTTCCTCACC | 923-942 |  |
| *Des2* | NM_027299.5 | F | GGTTACCACATGGAACACCA | 833-852 | 60 |
|  |  | R | GCAATCTTCCGCACCAGT | 886-903 |  |
| *Fa2h* | NM_178086.3 | F | TCACAGACACTCCGACAACG | 267-286 | 20 |
|  |  | R | CCATTCTCTGTGGGATCCTG | 337-356 |  |
| *Sptlc1* | NM_009269.2 | F | CTCAGGCACGGTACTTGGAC | 1292-1311 | 25 |
|  |  | R | CAGTGACCACAACCCTGATG | 1341-1360 |  |
| *Sptlc2* | NM_011479.4 | F | TCGGACAAGAAGACCCTGGA | 1080-1100 | 97 |
|  |  | R | GGCGAACAATAGACCCCTCC | 1138-1157 |  |
| *Sptlc3* | NM_001356507.1 | F | AAAGGCAAAGCACTGGTTGT | 361-380 | 107 |
|  |  | R | ATCCTCAGCATATGCCTTCC | 418-437 |  |
| *Sptssa* | NM_134054.2 | F | ATGGAAGCAGATGTCCTGGT | 116-135 | 62 |
|  |  | R | GGAAACCAGCATCGAATTG | 200-218 |  |
| *Sptssb* | NM_133675.3 | F | AGCATCACCGAGTACCAAGC | 122-141 | 33 |
|  |  | R | CCTTCACGCGCTTGAAATCC | 191-210 |  |
| *Cers1* | NM_138647.3 | F | GGTACCACAACGTAGGCCTC | 675-694 | 4 |
|  |  | R | TTGTGAACTCCAGCTGCACA | 724-743 |  |
| *Cers2* | NM_029789.2 | F | CCCTCATCATGGCTCTGCAT | 855-874 | 62 |
|  |  | R | GTTCTTCCATCCCGCGTAGT | 915-934 |  |
| *Cers3* | NM_001164201.1 | F | GGACTTCCTCGCTCATGTCA | 858-877 | 71 |
|  |  | R | GCACACCAGGAGAAGCTCAT | 904-923 |  |
| *Cers4* | NM_026058.4 | F | GCCCTTTGATGTCAAACGCA | 778-797 | 68 |
|  |  | R | GAAGCCTATCAGTCCCACGG | 834-853 |  |
| *Cers5* | NM_028015.2 | F | CCATGCCATCTGGTCCTACC | 1105-1124 | 38 |
|  |  | R | CCTTAGACACCTTTCCCCGG | 1156-1175 |  |
| *Cers6* | NM_001347161.2 | F | ACAATGTCACCTGGGCAGAC | 217-236 | 69 |
|  |  | R | AGGCAAGGTAGAGGTCCTCC | 272-291 |  |

(B) Human

| **Gene Name** | **Accession No.** |  | **Sequence (5'-3')** | **Position** | **Probe No.** |
| --- | --- | --- | --- | --- | --- |
| *DES1* | NM_003676.4 | F | GGAAGACTTCGAGTGGGTCTAC | 118-139 | 28 |
|  |  | R | TTCATCAAGGACTTTATCTCTGGA | 187-210 |  |
| *DES2* | NM_206918.3 | F | CGACTTCGAGTGGGTCTACA | 82-101 | 28 |
|  |  | R | TTGATGGCCGGGTACTTG | 142-159 |  |
| *FA2H* | NM_024306.5 | F | GGAGCAGTACTACGTGGGAGA | 296-316 | 32 |
|  |  | R | GCTACAGGCTCGTTCTCCAT | 342-361 |  |
| *SPTLC1* | NM_006415.4 | F | CATTAACTCAGGCGCGCTAC | 1317-1336 | 6 |
|  |  | R | GGACGCAGCTCTCTCCAGT | 1411-1429 |  |
| *SPTLC2* | NM_004863.4 | F | TGGATCCCGAGGATGTGGAT | 1152-1171 | 17 |
|  |  | R | AGGTGACAATGACGTGGCAT | 1278-1297 |  |
| *SPTLC3* | NM_018327.4 | F | GCAGAGCTTGGAAAAAGATTCTC | 1183-1205 | 38 |
|  |  | R | CAGATGCACGATGGAACCT | 1235-1253 |  |
| *SPTSSA* | NM_138288.4 | F | CCCCCATAGAGAACCACTAACA | 2053-2074 | 6 |
|  |  | R | TCAACTTCTCCTGCCTTCAAC | 2102-2122 |  |
| *SPTSSB* | NM_001320679.2 | F | CACAGGATCATTTCGTGAAGG | 121-141 | 41 |
|  |  | R | GCCCAGGAATGAGCACTAAG | 172-191 |  |
| *CERS1* | NM_021267.5 | F | GCCTTCCGGTACCACAATGT | 669-688 | 4 |
|  |  | R | AACTCAAGCTGCACGTCACT | 720-739 |  |
| *CERS2* | NM_022075.5 | F | TGGCTCTGCATGACTCTTCC | 925-944 | 109 |
|  |  | R | TGCAGGTGTTCTTCCATCCC | 983-1002 |  |
| *CERS3* | NM_178842.5 | F | TGGACGCAGACCTGTAACAC | 1153-1172 | 22 |
|  |  | R | GGAAAAACAATGAGGCGGCT | 1207-1226 |  |
| *CERS4* | NM_024552.3 | F | CCCCAGCTGACCAAGAAGTT | 662-681 | 78 |
|  |  | R | CCCACGAAGGAGGACAGGTA | 710-729 |  |
| *CERS5* | NM_147190.5 | F | GCAGGGACCCCTAAGCTTG | 87-105 | 76 |
|  |  | R | CGTTCTCGGGTAGCCAGAAG | 132-151 |  |
| *CERS6* | NM_203463.3 | F | CATTCAGCGCTGGTTTCGAC | 513-532 | 2 |
|  |  | R | TGCTCTCACAGAACCTCGTC | 564-583 |  |
| *GAPDH* | NM_002046.7 | F | AGCCACATCGCTCAGACAC | 57-75 | 60 |
|  |  | R | GCCCAATACGACCAAATCC | 104-122 |  |

**Supplementary Table 2**

MRM transitions for GSLs in mouse and human urinary bladder

| Ion mode | Compomd | Ceremide | Formula | Q1 | Q3 | CE (eV) | Internal standard |
| --- | --- | --- | --- | --- | --- | --- | --- |
| Positive | HexCer | d18:1/16:0-d3 | C40H74D3NO8 | 703.5916 | 264.27 | -40 | GlcCer d18:1/16:0-d3 |
|  |  | d18:1/h16:0 | C40H77NO9 | 716.5676 | 264.27 | -40 |  |
|  |  | d18:1/h20:0 | C44H85NO9 | 772.6302 | 264.27 | -40 |  |
|  |  | d18:1/h22:0 | C46H89NO9 | 800.6615 | 264.27 | -40 |  |
|  |  | d18:1/h23:0 | C47H91NO9 | 814.6772 | 264.27 | -40 |  |
|  |  | d18:1/h24:1 | C48H91NO9 | 826.6772 | 264.27 | -40 |  |
|  |  | d18:1/h24:0 | C48H93NO9 | 828.6928 | 264.27 | -40 |  |
|  |  | d18:1/16:0 | C40H77NO8 | 700.5727 | 264.27 | -40 |  |
|  |  | d18:1/22:0 | C46H89NO8 | 784.6666 | 264.27 | -40 |  |
|  |  | d18:1/24:1 | C48H91NO8 | 810.6822 | 264.27 | -40 |  |
|  |  | d18:1/24:0 | C48H93NO8 | 812.6979 | 264.27 | -40 |  |
|  |  | t18:0/24:0 | C48H95NO9 | 830.7085 | 282.28 | -40 |  |
|  |  | t18:0/h16:0 | C40H79NO10 | 734.5782 | 282.28 | -40 |  |
|  |  | t18:0/h18:0 | C42H83NO10 | 762.6095 | 282.28 | -40 |  |
|  |  | t18:0/h20:0 | C44H87NO10 | 790.6408 | 282.28 | -40 |  |
|  |  | t18:0/h22:0 | C46H91NO10 | 818.6721 | 282.28 | -40 |  |
|  |  | t18:0/h23:0 | C47H93NO10 | 832.6877 | 282.28 | -40 |  |
|  |  | t18:0/h24:1 | C48H93NO10 | 844.6877 | 282.28 | -40 |  |
|  |  | t18:0/h24:0 | C48H95NO10 | 846.7034 | 282.28 | -40 |  |
|  |  | d20:1/h24:0 | C50H97NO9 | 856.7241 | 292.30 | -40 |  |
|  |  | t20:0/24:0 | C50H99NO9 | 858.7398 | 310.31 | -40 |  |
|  |  | t20:0/h24:1 | C50H97NO10 | 872.7190 | 310.31 | -40 |  |
|  |  | t20:0/h24:0 | C50H99NO10 | 874.7347 | 310.31 | -40 |  |
|  |  | t20:0/h26:0 | C52H103NO10 | 902.7660 | 310.31 | -40 |  |
|  | LacCer | d18:1/17:0 | C47H89NO13 | 876.6412 | 264.27 | -40 | LacCer d18:1/17:0 |
|  |  | d18:1/16:0 | C46H87NO13 | 862.6255 | 264.27 | -40 |  |
|  |  | d18:1/22:0 | C52H99NO13 | 946.7194 | 264.27 | -40 |  |
|  |  | d18:1/24:1 | C54H101NO13 | 972.7351 | 264.27 | -40 |  |
|  |  | d18:1/24:0 | C54H103NO13 | 974.7507 | 264.27 | -40 |  |
|  |  | t18:0/h16:0 | C46H89NO15 | 896.6310 | 282.28 | -40 |  |
|  |  | t18:0/h22:0 | C52H101NO15 | 980.7249 | 282.28 | -40 |  |
|  |  | t18:0/h23:0 | C53H103NO15 | 994.7405 | 282.28 | -40 |  |
|  |  | t18:0/h24:0 | C54H105NO15 | 1008.7562 | 282.28 | -40 |  |
| Positive | Gb3Cer | d18:1/16:0. | C52H97NO18 | 1024.6783 | 264.27 | -50 | LacCer d18:1/17:0 |
|  |  | d18:1/20:0 | C56H105NO18 | 1080.7409 | 264.27 | -50 |  |
|  |  | d18:1/22:0 | C58H109NO18 | 1108.7722 | 264.27 | -50 |  |
|  |  | d18:1/24:1 | C60H111NO18 | 1134.7879 | 264.27 | -50 |  |
|  |  | d18:1/24:0 | C60H113NO18 | 1136.8035 | 264.27 | -50 |  |
|  | Gb4Cer | d18:1/17:0 | C47H89NO13 | 876.6412 | 264.27 | -70 | LacCer d18:1/17:0 |
|  |  | d18:0/20:0 | C64H120N2O23 | 1285.8360 | 266.28 | -70 |  |
|  |  | d18:1/16:0 | C60H110N2O23 | 1227.7577 | 264.27 | -70 |  |
|  |  | d18:1/18:0 | C62H114N2O23 | 1255.7890 | 264.27 | -70 |  |
|  |  | d18:1/20:0 | C64H118N2O23 | 1283.8203 | 264.27 | -70 |  |
|  |  | d18:1/22:0 | C66H122N2O23 | 1311.8516 | 264.27 | -70 |  |
|  |  | d18:1/23:0 | C67H124N2O23 | 1325.8673 | 264.27 | -70 |  |
|  |  | d18:1/24:1 | C68H124N2O23 | 1337.8673 | 264.27 | -70 |  |
|  |  | d18:1/24:0 | C68H126N2O23 | 1339.8829 | 264.27 | -70 |  |
| Negative | GM3 | d18:1/18:0-d3 | C59H104D3N2O21 | 1180.7480 | 290.09 | 50 | GM3 d18:1/18:0-d3 |
|  |  | d18:1/16:0 | C57H104N2O21 | 1151.7053 | 290.09 | 50 |  |
|  |  | d18:1/22:0 | C63H116N2O21 | 1235.7992 | 290.09 | 50 |  |
|  |  | d18:1/24:1 | C65H118N2O21 | 1261.8148 | 290.09 | 50 |  |
|  |  | d18:1/24:0 | C65H120N2O21 | 1263.8305 | 290.09 | 50 |  |
|  |  | t18:0/h22:0 | C63H118N2O23 | 1269.8047 | 290.09 | 50 |  |
|  |  | t18:0/h23:0 | C64H120N2O23 | 1283.8203 | 290.09 | 50 |  |
|  |  | t18:0/h24:1 | C65H120N2O23 | 1295.8203 | 290.09 | 50 |  |
|  |  | t18:0/h24:0 | C65H122N2O23 | 1297.8360 | 290.09 | 50 |  |
|  |  | t20:0/h24:1 | C67H124N2O23 | 1323.8516 | 290.09 | 50 |  |
|  |  | t20:0/h24:0 | C67H126N2O23 | 1325.8673 | 290.09 | 50 |  |
|  |  | t20:0/h26:0 | C69H130N2O23 | 1353.8986 | 290.09 | 50 |  |
|  | GD3 | d18:1/18:0-d3 | C70H122D3N3O29 | 736.4215 | 290.09 | 30 | GD3 d18:1/18:0-d3 |
|  |  | d18:1/24:1 | C76H135N3O29 | 775.9512 | 290.09 | 30 |  |
|  |  | d18:1/24:0 | C76H137N3O29 | 776.9590 | 290.09 | 30 |  |
|  |  | t18:0/h24:1 | C76H137N3O31 | 792.9539 | 290.09 | 30 |  |
|  |  | t20:0/h24:0 | C78H143N3O31 | 807.9774 | 290.09 | 30 |  |
